# What makes (hydroxy)chloroquine ineffective against COVID-19: insights from cell biology

**DOI:** 10.1093/jmcb/mjab016

**Published:** 2021-03-09

**Authors:** Dania Altulea, Sjors Maassen, Maksim V Baranov, G van den Bogaart

**Affiliations:** 1 Department of Molecular Immunology and Microbiology, Groningen Biomolecular Sciences and Biotechnology Institute, University of Groningen, Groningen, the Netherlands; 2 Department of Tumor Immunology, Radboud Institute for Molecular Life Sciences, Radboud University Medical Center, Nijmegen, the Netherlands

**Keywords:** coronavirus, COVID-19, SARS-CoV-2, chloroquine, hydroxychloroquine

## Abstract

Since chloroquine (CQ) and hydroxychloroquine (HCQ) can inhibit the invasion and proliferation of severe acute respiratory syndrome coronavirus 2 (SARS-CoV-2) in cultured cells, the repurposing of these antimalarial drugs was considered a promising strategy for treatment and prevention of coronavirus disease (COVID-19). However, despite promising preliminary findings, many clinical trials showed neither significant therapeutic nor prophylactic benefits of CQ and HCQ against COVID-19. Here, we aim to answer the question of why these drugs are not effective against the disease by examining the cellular working mechanisms of CQ and HCQ in prevention of SARS-CoV-2 infections.

## Introduction

**Figure 1:**
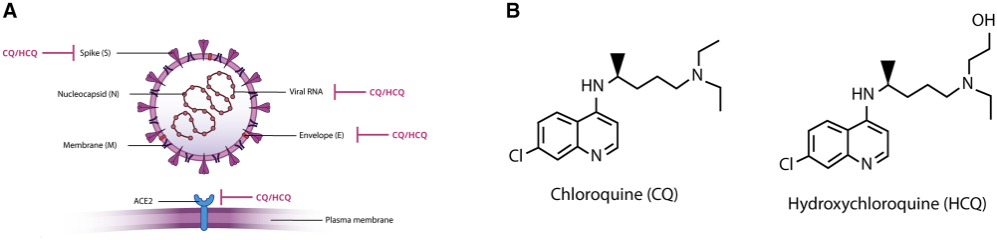
Direct binding of CQ and HCQ to SARS-CoV-2. (**A**) The nucleocapsid protein (N) forms complexes with the viral RNA, which is positive-sense and single-stranded, and interacts with the membrane protein (M) during viral replication and assembly. The spike protein (S) facilitates viral entry and invasion of the host cells by interacting with the receptor ACE2. Finally, the envelope protein (E) is a glycoprotein that plays a role in viral assembly and release. CQ and HCQ are shown or proposed to bind to viral RNA (Kuznik et al., 2011), S protein (Bibi et al., 2020), E protein (Gentile et al., 2020), and ACE2 (Wang et al., 2020b). (**B**) The structures of CQ and its hydroxylated analogue HCQ. Shown are the more bioactive S-enantiomers.

The severe acute respiratory syndrome coronavirus 2 (SARS-CoV-2; Figure 1A), the cause of coronavirus disease (COVID-19), belongs to the *Coronaviridae* family of viruses, as do SARS-CoV and the Middle East respiratory syndrome coronavirus (MERS-CoV), which, respectively, caused pandemics in 2002‒2003 and 2012 (Coronaviridae Study Group of the International Committee on Taxonomy of Viruses, 2020). Two candidate drugs for treatment and prevention of COVID-19 are the antimalarial drugs chloroquine (CQ) and hydroxychloroquine (HCQ) (Schluenz et al., 2020; Sun et al., 2020; Infante et al., 2021). CQ is the synthetic form of quinine, a natural alkaloid extracted from the barks of the cinchona trees that are native to Peru (Figure 1B). HCQ is the more soluble and less toxic derivative of CQ (Plantone and Koudriavtseva, 2018). The main advantages of CQ and HCQ are that they are generally well-tolerated, affordable, readily available, and have been prescribed for a long time, and thus their toxicity and pharmacology are well-documented (Zhong et al., 2020). They are widely used to treat malaria. Additionally, HCQ is also chronically used to treat patients with autoimmune diseases such as systemic lupus erythematosus (SLE), Sjögren's syndrome, and rheumatoid arthritis (Vesterinen et al., 2015; Ferreira et al., 2021). 

The antiviral activity of HCQ and CQ has been known for quite a long time, since these drugs have been shown to inhibit invasion of different viruses in cultured cells *in vitro*, including human immunodeficiency virus (HIV), influenza virus, Zika virus, and dengue virus, although most animal studies showed that these compounds are not very efficient in preventing viral infections *in vivo* (Hashem et al., 2020). Prior to the SARS-CoV-2 pandemic, researchers also demonstrated the ability of CQ and HCQ to inhibit the viral replication of members of the *Coronaviridae*, including SARS-CoV, MERS-CoV, and human coronavirus OC43 (HCoV-OC43), in cell lines with the half-maximal effective concentration (EC_50_) mostly between 1 and 10 µM. However, *in vivo* studies in animal models again showed more ambiguous results (Hashem et al., 2020; Pastick et al., 2020). For instance, CQ at a dose of 15 mg/kg body weight could prevent HCoV-OC43 infection in mice (Keyaerts et al., 2009), but doses between 1 and 50 mg/kg were ineffective against SARS-CoV (Barnard et al., 2006).

Given the ability of CQ and HCQ to block invasion of cultured cells by other members of the *Coronaviridae*, it is not surprising that a multidrug screen identified these compounds as being capable of reducing SARS-CoV-2 infections *in vitro* (IC_50_ of CQ: 42.0–56.8 µM; IC_50_ of HCQ: 9.2–11.2 µM) (Weston et al., 2020). In fact, it is now firmly established that both CQ and HCQ can limit SARS-CoV-2 invasion and proliferation in cell culture, although the reported effective concentrations vary by almost two orders of magnitude among studies (EC_50_ between 0.7 and 50 µM). Overall, the ability of CQ and HCQ to block invasion of mammalian cells depends on the cell line, viral strain, and route of entry (Hoffmann et al., 2020b), as to be discussed below. Not only does HCQ inhibit host cell invasion by SARS-CoV-2, but pulse-chase experiments demonstrated that HCQ could also impact later stages of the viral life cycle (Weston et al., 2020).

These *in vitro* findings suggested that CQ and HCQ would be effective for treatment and prevention of COVID-19; a hypothesis supported by several early clinical observations. For example, analysis of COVID-19 testing results in Portugal found that patients suffering from autoimmune diseases who chronically took HCQ had a lower rate of infection than individuals who did not take HCQ (Ferreira et al., 2021). Unfortunately, although early retrospective clinical trials suggested efficacy and several clinical trials are still ongoing, it is becoming clear from systematic review and meta-analyses of clinical trials that CQ and HCQ are not sufficiently effective against COVID-19 (Ayele Mega et al., 2020; Kashour et al., 2021). Particularly important is that several large-scale, randomized trials addressing the efficacy of CQ and HCQ were stopped early due to lack of efficacy: the RECOVERY trial in the UK, the ORCHID trial in the USA, and the SOLIDARITY trial from the World Health Organization (RECOVERY Collaborative Group et al., 2020; Self et al., 2020; WHO Solidarity Trial Consortium et al., 2020). Recent experiments in macaques, non-human primates, also showed no effect on viral load in any of the analyzed tissues regardless of whether HCQ was administered before or after peak viral load (Maisonnasse et al., 2020). Moreover, HCQ did not confer protection against infection with SARS-CoV-2 when the drug was used as a pre-exposure prophylaxis treatment (Maisonnasse et al., 2020). The goal of this review is to discuss the molecular mechanisms of treatment and prevention of SARS-CoV-2 infection by HCQ and CQ in order to identify the reasons why *in vitro* findings did not correlate with *in vivo* findings in animal studies and in the clinic.

## CQ and HCQ block cell invasion of SARS-CoV-2 *in vitro*

**Figure 2:**
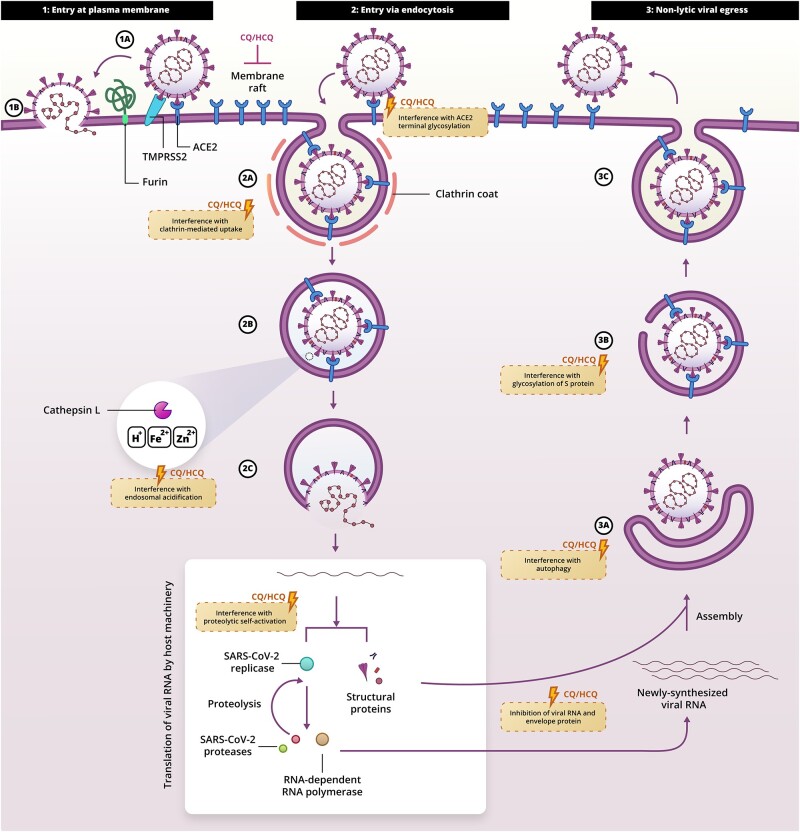
SARS-CoV-2 host cell invasion and egress pathways. The virus invades the cells after binding to the ACE2 receptor either via the cell membrane (step 1A) or via an endosomal pathway (2A and 2B). Both pathways result in the release of the viral RNA in the cytoplasm of the host cell (1B and 2C). CQ and HCQ might hinder viral entry into the cell by interference with endosomal acidification (blocking activation of cathepsin L and iron import), zinc sequestration, interference with ACE2 terminal glycosylation, interference with proteolytic self-activation of furin, and blockage of clathrin-mediated endocytosis and/or blockage of uptake via lipid rafts. CQ and HCQ might block viral proliferation by interference with glycosylation of S protein (3B) and/or interference with autophagy (3A).

SARS-CoV-2 is primarily transmitted directly through inhalation of contaminated respiratory droplets (Li et al., 2020b). Subsequently, the viral particles bind to the epithelium of the respiratory tract by high-affinity interactions of the spike protein (S) of SARS-CoV-2 with the angiotensin-converting enzyme 2 (ACE2) receptor on the surface of epithelial cells (Vabret et al., 2020). For host cell invasion, the S protein needs to be activated by a two-step sequential proteolytic cleavage: first a priming cleavage between the N-terminal S1-region and the C-terminal S2-region and then an activating cleavage on the S2-region (Ou et al., 2020). The priming cleavage is done by furin, a protease primarily present in the Golgi network but also at the plasma membrane and in recycling endosomes (Teuchert et al., 1999; Braun and Sauter, 2019), which cleaves at a region between the S1 and S2 subunits containing several positively charged arginine residues (681-PRR-683) (Figure 2, step 1A; Hoffmann et al., 2020a).

Following binding to ACE2, SARS-CoV-2 can invade the host cells in two ways: the plasma membrane and the endocytosis pathways (Figure 2; Shang et al., 2020; South et al., 2020; Tang et al., 2020). In the endocytosis pathway, the binding of the S protein to ACE2 results in the uptake of the ACE2‒virus complex into an endosome, where the second activation cleavage of the S protein can be mediated by cathepsin L (695-YT-696) (Figure 2, steps 2A and 2B; Simmons et al., 2005; Bosch et al., 2008; Ou et al., 2020). The activated S protein then mediates fusion of the viral membrane with the endosomal membrane, eventually leading to the release of the viral RNA into the cytosol (Figure 2, step 2C; Ou et al., 2020). In the pathway of invasion via the plasma membrane, the S protein becomes activated on the host cell surface by a type II transmembrane serine protease (TMPRSS2; 685-RS-686) or related proteins (e.g. TMPRSS4, TMPRSS11A, TMPRSS11D, and TMPRSS11E), and this results in direct fusion of the viral membrane with the plasma membrane (Figure 2, steps 1A and 1B; Ou et al., 2020).

For SARS-CoV, experiments with caveolin-1-negative HepG2 cells, the clathrin-mediated endocytosis inhibitor chlorpromazine, and small interfering RNA-mediated gene silencing for the clathrin heavy chain showed that its invasion of host cells occurs primarily via clathrin-mediated endocytosis (Figure 2, step 2A; Inoue et al., 2007). However, for SARS-CoV-2, the preferred host cell invasion pathway is less clear and probably cell type-dependent. Experiments with the lysosomotropic compounds ammonia chloride and bafilomycin A, which lead to a more basic endo-lysosomal pH, and with an inhibitor for PIKfyve, a phosphoinositide kinase needed for the conversion of endosomes into lysosomes (Baranov et al., 2019), showed that SARS-CoV-2 predominantly invades HEK293 cells recombinantly expressing human ACE2 via the endocytosis pathway (Ou et al., 2020). However, findings from a preliminary study suggest that the preferred invasion pathway of SARS-CoV-2 in several cultured cell types is via the plasma membrane, because TMPRSS2 inhibitors efficiently blocked invasion by SARS-CoV-2 whereas cathepsin inhibitors blocked it less efficiently (Figure 2, step 2B; Zhu et al., 2020). Although these findings have to be interpreted with caution as this study deposited at the preprint repository bioRxiv still needs to undergo peer review, this study also suggested that preferential entry depends on the polybasic furin cleavage site (681-PRR-683) between the S1-region and S2-region, which is lacking in the S proteins of SARS-CoV and MERS-CoV (Zhu et al., 2020). These contrasting findings concerning the predominant pathway of cell invasion might be caused by different viral strains of SARS-CoV-2 employing different preferential cellular invasion pathways, as the polybasic cleavage site seems unstable and deletion variants have been found in cell culture and in patient samples (Lau et al., 2020; Liu et al., 2020b; Wong et al., 2020).

Given that the pathway of infection depends on the cell type and viral strain, CQ and HCQ need to block both mechanisms of cell entry in order for these drugs to be effective in blocking infections of SARS-CoV-2. However, recent evidence suggests that this might not be the case, and CQ and HCQ might not efficiently block the plasma membrane pathway of cell invasion: while CQ and HCQ blocked infection in Vero E6 cell line (a kidney epithelial cell line that expresses ACE2 but not TMPRSS2; IC_50_ of CQ: 6.5 µM; IC_50_ of HCQ: 13.3 µM), these drugs did not block infection in Vero E6 cells recombinantly expressing TMPRSS2 (Hoffmann et al., 2020b). Moreover, the effective dose for inhibiting SARS-CoV-2 invasion of Calu-2 cells, a lung epithelial cell line expressing TMPRSS2 endogenously, was an order of magnitude higher (IC_50_ of CQ: 64.7 µM; IC_50_ of HCQ: 119 µM) than for Vero E6 cells (Hoffmann et al., 2020b).

The mechanism of action of how CQ and HCQ block viral entry and progression is still not completely understood, with several mechanisms being proposed: blocking of endosomal acidification, interference with glycosylation of ACE2 or viral proteins, direct binding to ACE2 or viral proteins, interference with viral endocytosis, sequestering of metals, and exertion of immunomodulatory effects.

## Blocking of endosomal acidification by CQ and HCQ

The antiviral activity of CQ and HCQ has mostly been attributed to their passive diffusion into acidic cellular compartments, such as endosomes, lysosomes, and the Golgi network (Krogstad and Schlesinger, 1987; Yoon et al., 2010). CQ and HCQ are weak lysosomotropic bases that can bind to two protons (albeit with relatively high pKa: pKa_1_ = 8.1, pKa_2_ = 10.2) and thus counteract the activity of the vacuolar-ATPase (Al-Bari, 2015). This protonation makes HCQ and CQ more hydrophilic, and these compounds thereby not only increase the pH but can also be accumulated 100–1000-fold in the lumen of lysosomes causing osmotic swelling (Ohkuma and Poole, 1981; Kaufmann and Krise, 2007). Because of this reduced acidification and/or osmotic swelling, CQ and HCQ at concentrations of ∼10–100 µM are well known to disrupt endosomal function (Figure 2, step 2B; Sundelin and Terman, 2002; Mauthe et al., 2018). The accumulation of CQ and HCQ in the food vacuoles of the malaria parasite *Plasmodium* and the subsequent prevention of acidification of this compartment underlie the efficacy of CQ and HCQ as anti-malaria drugs (Kaur et al., 2010). The reduced acidification might also be the reason for prevention of SARS-CoV-2 invasion via the cathepsin L-dependent endocytic pathway: since cathepsin L has an acidic pH optimum (around pH 5.5) and is unstable at higher pH (Turk et al., 1993), CQ and HCQ could lower the activity of these proteases and block proteolytic activation of the S protein and thus block subsequent fusion between the viral envelope and lysosomal or endosomal membranes.

For several reasons, the alkalization of organelles by CQ and HCQ might also inhibit the invasion of SARS-CoV-2 at the plasma membrane. First, the acidification of the Golgi network is important for protein glycosylation, which will be explained in the next session. Second, many newly synthesized proteases are proteolytically activated while in transit to the plasma membrane in the Golgi network or a post-Golgi compartment. For example, furin, required for pre-processing of the viral S protein (Figure 2, step 1A; Hoffmann et al., 2020a), is synthesized in the endoplasmic reticulum with an auto-inhibitory fragment that needs to be proteolytically cleaved off in the trans-Golgi network in a pH-dependent manner (Anderson et al., 1997). By interfering with organellar acidification, CQ and HCQ might reduce activation of furin.

It is also possible that the blockage of acidification affects viral propagation during postinvasion phases of the viral cycle of SARS-CoV-2 by impairing its replication, assembly, or trafficking. The inhibition of lysosomal acidification by HCQ and CQ is well known to block the fusion of lysosomes with autophagosomes, and thus concentrations of ∼50–100 µM effectively inhibit autophagy in cell culture (Carew et al., 2011; Mauthe et al., 2018). Many viruses utilize components of the autophagic machinery for their intracellular propagation or for non-lytic cellular egress (Kudchodkar and Levine, 2009). There is also evidence that the autophagy pathway plays a role in the replication cycle of members of the *Coronaviridae*, including MERS-CoV (Gassen et al., 2019), and that blockage of autophagosome‒lysosome fusion in infected cells causes accumulation of autophagosomes, which in turn triggers an apoptotic pathway and disrupts the virus replication cycle (Figure 2, step 3A; Shojaei et al., 2020; Yang and Shen, 2020).

Finally, the acidification might interfere with the iron uptake by host cells. Many of the steps in cellular iron uptake, such as the dissociation of Fe^3+^ from transferrin and the export of iron from the lumen of endosomes into the cytosol, are dependent on the organellar pH and could therefore be distorted by CQ and HCQ as has recently been proposed (Quiros Roldan et al., 2020). In cultured embryonic fibroblasts, 100 µM CQ was shown to inhibit transferrin uptake (Octave et al., 1982). Thus, CQ and HCQ might induce cellular iron starvation, inhibit the viral life cycle, and/or dampen immunity (Figure 2, step 2B; Quiros Roldan et al., 2020).

## Interference of CQ and HCQ in glycosylation of ACE2 or viral proteins

CQ at concentrations of ∼25 µM affects the terminal glycosylation of the ACE2 receptor (trimming of the N-glycosylated chains), thereby lowering its binding efficiency to the S protein of SARS-CoV-2 (Vincent et al., 2005). Since correct Golgi acidification is required for correct organization of the Golgi network and for glycosylation (Anderson et al., 1997; Linders et al., 2020), this blockage of glycosylation is a possible consequence of the reduced acidification of the Golgi network (Figure 2, step 2). Indeed, CQ can result in visible disruption of the Golgi network (at 100 µM concentration) (Mauthe et al., 2018), and the drug-mediated pH increase might cause a re-localization of the glycosyltransferases from the Golgi apparatus to other acidic compartments, leading to a defective glycosylation process (Axelsson et al., 2001).

Alternatively, or additionally, CQ and HCQ might directly block the function of proteins involved in glycosylation, such as the proteins that synthesize the monosaccharide precursors, as has recently been proposed (Savarino et al., 2006). Even at a low concentration of 1 µM, CQ is capable of blocking the activity of purified ribosyldihydronicotinamide dehydrogenase (NQO2) (Kwiek et al., 2004), and has therefore been proposed to inhibit structurally homologous enzymes involved in the biosynthesis of sialic acids (Savarino et al., 2006), one of the monosaccharide precursors for glycosylation involved in cellular invasion of several coronaviruses (Matrosovich et al., 2015). However, this hypothesis needs to be addressed experimentally.

In addition to ACE2, structure‒function studies suggest that the S protein of SARS-CoV-2 is also highly glycosylated (Walls et al., 2020), possibly to regulate its stability, trafficking, and/or function, and this glycosylation might also be disrupted by CQ and HCQ. Indeed, CQ was shown to affect the biosynthesis and glycosylation of the S protein of SARS-CoV, albeit at high concentrations of 100 µM (Figure 2, step 3B; Vincent et al., 2005). Finally, the glycosylation in the Golgi network is not only required for the synthesis of glycoproteins but also for glycolipids such as gangliosides. Structural modelling suggested that the S protein of SARS-CoV-2 might bind to sialic acids linked to gangliosides (Fantini et al., 2020) and this might facilitate host cell invasion as shown for other coronaviruses (Matrosovich et al., 2015), although these theoretical predictions need experimental validation. By interfering with the biosynthesis of both glycoproteins and glycolipids, CQ and HCQ might thus interfere with the binding of the viral S protein to the host cell.

## Direct binding of CQ and HCQ to ACE2 or viral proteins

Not only can CQ and HCQ block interactions of the S protein with ACE2 by disturbing the glycosylation of one or both of these proteins, but also by direct competitive binding. Surface plasmon resonance experiments with purified proteins showed that binding of both CQ and HCQ to the ACE2 protein, with a K_d_ of 0.7 µM for CQ and 0.4 µM for HCQ, correlated with blockage of viral invasion of HEK293T cells expressing human ACE2 (Wang et al., 2020b). Alternatively, CQ might bind to the S protein as suggested by molecular docking and molecular dynamics simulations (Bibi et al., 2020), although these theoretical predictions need experimental validation. CQ and HCQ might thus potentially intervene with viral invasion by competitive binding to the host cell receptor ACE2 and/or the viral S protein (Figure 1A).

In addition to the S protein, CQ and HCQ have been proposed to interact with several other viral proteins. M^pro^ is one of the 12 bioactive fragments produced by autocleavage of the viral replicase polyprotein 1ab (Rep) and is required for activation of the viral replicase (Li and Kang, 2020). Molecular docking and enzymatic inhibition studies with purified proteins showed that M^pro^ binds in an enantioselective manner to the S-enantiomer of CQ and HCQ with a relatively high affinity (IC_50_ value of 2.5 µM for HCQ) (Belhassan et al., 2020; da Silva Arouche et al., 2020). A recently submitted study to the preprint server bioRxiv confirmed that the S-enantiomers of CQ and HCQ are ∼2-fold more effective in blocking SARS-CoV-2 invasion of cultured Vero E6 cells (Li et al., 2020a), although this study still needs to undergo peer review. Based on molecular docking, CQ and HCQ have also been predicted to interact with the E protein, necessary in the maturation processes of the virus, and two other cleavage products of Rep guanine-N7 methyltransferase (nsp10/nsp14) and 2′-O-methyltransferase (nsp10/nsp16), involved in proofreading and capping of viral RNA (Gentile et al., 2020). However, these interactions have not been confirmed experimentally.

## Interference of CQ and HCQ with viral endocytosis

As mentioned above, SARS-CoV is internalized via clathrin-mediated endocytosis following the binding of its S protein to the ACE2 receptor at the host cell membrane (Inoue et al., 2007). Several studies suggest that CQ and HCQ might interfere with this host cell endocytosis (Figure 2, step 2A). CQ at a concentration of 60 µM caused reduced internalization of fluorescently labelled dextran by retinal pigment epithelial cells (Chen et al., 2011). In another study, CQ at concentrations >20 µM was found to inhibit the uptake of nanoparticles and liposomes by various macrophage and cancer cell lines (Wolfram et al., 2017). The mechanism of how CQ affects endocytosis is incompletely understood, but mass spectrometry revealed that 100 µM CQ changed the levels of several cytoskeletal and ribosomal proteins, including a reduction of phosphatidylinositol binding clathrin assembly protein (PICALM), which plays a critical role in clathrin-mediated endocytosis (Wolfram et al., 2017).

CQ and HCQ might also block non-clathrin-dependent forms of endocytosis that depend on so-called lipid rafts at the plasma membrane, such as caveolin-mediated endocytosis (Lajoie and Nabi, 2010). Lipid rafts are transient assemblies of cholesterol, gangliosides, and other sphingolipids that can cluster or segregate specific proteins, thereby functioning in membrane signalling and endocytosis (Lingwood and Simons, 2010). It was found by super-resolution microscopy on HEK293T cells overexpressing human ACE2 that 50 µM HCQ distorts lipid rafts (Figure 2, step 2), possibly by inserting in the membrane and affecting the membrane packing (Yuan et al., 2020). This was proposed to inhibit the endocytosis of ACE2, as the disruption of lipid rafts depended on the recruitment of ACE2 to these lipid rafts (Yuan et al., 2020). Therefore, HCQ might distort the internalization of the virus together with ACE2 (Yuan et al., 2020). Supporting the notion that CQ and HCQ might disrupt lipid rafts comes from molecular modelling suggesting that CQ and HCQ might directly bind to gangliosides and thus interfere with the binding of the viral S protein to the host cell (Fantini et al., 2020), although these theoretical predictions need to be experimentally confirmed.

## Sequestering of metals by CQ and HCQ

The final proposed mechanism of how CQ and HCQ can block host cell invasion of SARS-CoV-2 involves the sequestration of metal cations. CQ (and presumably also HCQ) can bind to Zn^2+^ and CQ at concentrations of ∼100 µM enhances the zinc uptake in cell cultures and sequesters Zn^2+^ in lysosomes (Figure 2, step 2B; Xue et al., 2014). For SARS-CoV, it was found that zinc inhibits the viral RNA-dependent RNA polymerase (nsp12) *in vitro* (te Velthuis et al., 2010). Low concentrations of a well-known zinc-ionophore (2 µM pyrithione) with zinc were found to be effective in blocking invasion of SARS-CoV in Vero E6 cells (te Velthuis et al., 2010).

## Immunosuppressive effects of CQ and HCQ

In addition to these direct antiviral effects, CQ and HCQ also possess anti-inflammatory and immune regulatory properties (Schrezenmeier et al., 2020; Sun et al., 2020; Vabret et al., 2020). These immunosuppressive effects of CQ and HCQ might be beneficial for COVID-19 patients, as they limit the detrimental immune response that is responsible for much of the lethality of COVID-19 (Mehta et al., 2020; Zhong et al., 2020). However, they might also aggravate COVID-19, as they prevent an effective immune response and therefore might prevent effective immune clearance of the virus (Sun et al., 2020).

A main mechanism by which HCQ and CQ dampen the immune response is likely related to their inhibition of lysosomal acidification, as it has been proposed that this interferes with the interaction of viral RNA with pathogen recognition receptors such as Toll-like receptors (TLRs) TLR-7 and TLR-8 (Hashem et al., 2020). In line with this, CQ at concentrations >1 µM has been shown to block recognition of microbial DNA patterns by TLR-9 in endosomes (Häcker et al., 1998). This inhibition might be caused by the impaired activation of TLR-9, because it needs to be proteolytically activated in the lumen of acidic endosomes (Ewald et al., 2008). Alternatively, spectroscopic measurements revealed direct binding of CQ to nucleic acids, and it has been proposed that CQ might therefore directly bind to the viral RNA in the lumen of endosomes and mask the recognition of the RNA by TLRs (Kuznik et al., 2011).

CQ and HCQ also exert immunomodulatory effects downstream of pathogen recognition (Infante et al., 2021). For example, 25–100 µM CQ and HCQ were shown to reduce the production of the proinflammatory cytokine interleukin 1β (IL-1β) in LPS-stimulated human peripheral blood mononuclear cells (Jang et al., 2006) and 0.3–300 µM CQ and HCQ decreased the secretion of the proinflammatory cytokines IL-6 and tumor necrosis factor-α (TNF-α) in *in vitro* cultured monocytes (Picot et al., 1991). However, in a macaque study, HCQ treatment did not result in immunosuppressive effects and did not prevent lymphocytopenia nor pulmonary lesions (Maisonnasse et al., 2020). In contrast, HCQ with a loading dose of 90 mg/kg body weight followed by a daily maintenance dose of 45 mg/kg resulted in a significant increase in plasma levels of TNF-α 2 days postinfection, whereas the levels of anti-inflammatory IL-1 receptor antagonist (IL-1RA) were reduced (Maisonnasse et al., 2020).

## HCQ and CQ concentrations in clinical trials

The dosages of CQ and HCQ used in clinical trials are based on their effective concentrations to prevent SARS-CoV-2 infection *in vitro* and on the pharmacokinetics and pharmacodynamics of these drugs (Al-Kofahi et al., 2020; Yao et al., 2020). For example, based on modelling, a regimen was calculated to treat COVID-19 with HCQ consisting of a loading dose of 400 mg twice on the first day of diagnosis, followed by maintenance dosages of 200 mg twice daily for 4 days (Yao et al., 2020). This dosage is close to that recommended by the FDA for the prevention of malaria (a loading dose of 800 mg, followed by a dose of 400 mg/day for 3 days), even though the *in vitro* effectivity of CQ and HCQ for SARS-CoV-2 is >20-fold lower than that for malaria (Al-Kofahi et al., 2020). Similar dosages have been used in most clinical trials, with one or two loading doses of 800 mg followed by 400 mg each 6‒12 h by oral ingestion. However, preliminary findings from several studies showed that the concentrations of HCQ or CQ achieved in circulation of most subjects might not be sufficient to be effective. For instance, two studies revealed a concentration of HCQ only up to 1–3 µM in the blood in the period 0.1–8.5 h after oral dose (Marzolini et al., 2020; MacGowan et al., 2021). Similarly, a non-peer-reviewed study at medRxiv reported concentrations of CQ at ~1 µM in plasma of COVID-19 patients (Brüggemann et al., 2020). As these concentrations of HCQ and CQ are generally lower than required for prevention of viral invasion *in vitro* (Liu et al., 2020a; Wang et al., 2020a; Weston et al., 2020; Yao et al., 2020) and for its immunomodulatory effects (Picot et al., 1991; Häcker et al., 1998; Jang et al., 2006), they are likely too low to be pharmacologically active (Gonçalves et al., 2020). Thus, at least in circulation, the levels of CQ and HCQ achieved in clinical trials are likely insufficient to inhibit SARS-CoV-2 spread.

The difficulty to reach sufficiently high concentrations is probably caused by the lysosomotropic properties of CQ and HCQ. Because of these properties, CQ and HCQ are passively taken up by various types of cells and extensively sequestered in endo/lysosomes. Consequently, HCQ displays a long half-life in the body (∼40–60 days) and reaches steady-state concentrations in circulation very slowly due to the high sequestration in intracellular endosomal compartments (Schrezenmeier and Dörner, 2020; Infante et al., 2021). This slow drug accumulation also accounts for a delay in therapeutic effect, as is for instance well known in SLE where therapeutic effects can appear after weeks or even months following the initiation of the HCQ treatment (Ponticelli and Moroni, 2017). It might therefore be very well that the duration of the treatment of COVID-19 patients with CQ or HCQ is not long enough to reach effective concentrations. Although CQ and HCQ might still be used as a prophylactic measure, this implies that these drugs would need to be ingested for weeks or months before effective protection is achieved. The adverse side effects of these compounds might make this an unfeasible approach (Gonçalves et al., 2020; Ren et al., 2020).

On the other hand, while the concentrations of CQ and HCQ in the circulation might be too low to prevent viral spread, a study in macaques suggested that their concentrations in the lung, which is the primary site of infection, might actually be above the EC_50_ values of *in vitro* assays (Maisonnasse et al., 2020). In this study, a treatment regimen of 90 mg/kg body weight on the first day followed by a daily maintenance dose of 45 mg/kg, resulted in concentrations of HCQ in the lung much higher than in plasma (plasma: 0.3–1.2 µM; lung: 9–60 µM) (Maisonnasse et al., 2020). However, in this study, no anti-viral activity of HCQ was observed, regardless of whether the drug was used after infection or prophylactically (Maisonnasse et al., 2020). One possible cause of this discrepancy might be that whereas the overall concentration of HCQ in the lung could be quite high, most HCQ might still be accumulated in the endo/lysosomes of lung cells and the local concentration at the actual site of viral entry (i.e. the surface of the lung epithelium) might still be too low (Schrezenmeier and Dörner, 2020; Infante et al., 2021).

## Conclusion

**Figure 3:**
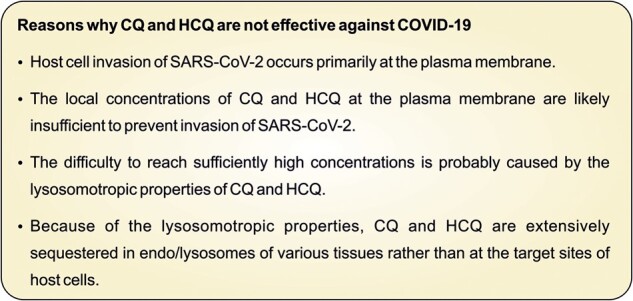
Reasons why CQ and HCQ are ineffective against COVID-19.

From clinical trials, the conclusion is emerging that CQ and HCQ offer no, or only a very limited, benefit for COVID-19 patients (RECOVERY Collaborative Group et al., 2020; Self et al., 2020; WHO Solidarity Trial Consortium et al., 2020). The cellular mechanism of how these drugs exert their antiviral and immunomodulatory actions is not well understood, with a wide variety of different mechanisms proposed. However, all proposed mechanisms require quite high (>5 µM) concentrations of CQ and HCQ. Since CQ and HCQ are lysosomotropic and sequestered in acidic organelles (Schrezenmeier and Dörner, 2020; Infante et al., 2021), safe therapeutic dosages of HCQ and CQ do likely not result in sufficient levels of CQ and HCQ in the circulation and likely also not at the surface of the lung epithelium. Since SARS-CoV-2 preferentially invades host cells at the plasma membrane (Hoffmann et al., 2020b) due to structural alterations in the proteolytic activation site of the S protein (Zhu et al., 2020), the achievable concentrations of CQ and HCQ at the prime site of infection might thus be too low to inhibit SARS-CoV-2 spread (Figure 3).

## Funding 

This work was supported by a Young Investigator Grant from the Human Frontier Science Program (HFSP; RGY0080/2018), a Vidi grant from the Netherlands Organization for Scientific Research (NWO-ALW VIDI 864.14.001), and funding from the European Research Council (ERC) under the European Union’s Horizon 2020 research and innovation programme (grant agreement no. 862137).

**Conflict of interest:** none declared. 
